# Predictors of Impaired Glucose Regulation in Patients with Non-Alcoholic Fatty Liver Disease

**DOI:** 10.1155/2012/351974

**Published:** 2011-09-21

**Authors:** Erifili Hatziagelaki, Drosos E. Karageorgopoulos, Athina Chounta, Anastasia Tsiavou, Matthew E. Falagas, George Dimitriadis

**Affiliations:** ^1^2nd Department of Internal Medicine, Research Institute and Diabetes Center, University of Athens Medical School, Attikon University Hospital, 12462 Athens, Greece; ^2^Alfa Institute of Biomedical Sciences (AIBS), Marousi, 15123 Athens, Greece; ^3^4th Department of Internal Medicine, University of Athens Medical School, Attikon University Hospital, 12462 Athens, Greece; ^4^Department of Medicine, Henry Dunant Hospital, 11526 Athens, Greece; ^5^Department of Medicine, Tufts University School of Medicine, Boston, MA 02111, USA

## Abstract

*Introduction*. Many patients with non-alcoholic fatty liver disease (NAFLD) have impaired glucose regulation or type 2 diabetes mellitus (DM). We investigated characteristics of NAFLD patients associated with hyperglycemia. *Methods*. During a 2-hour oral glucose tolerance test (OGTT), serum glucose and insulin were measured in 152 NAFLD patients. *Results*. 48.7% of NAFLD patients had hyperglycemia. Age (odds ratio (OR) = 1.08, 95% confidence interval (CI): 1.03–1.13), body mass index (BMI) (OR = 1.12, 95% CI: 1.01–1.25), and lower high-density lipoprotein cholesterol (HDL-C) (OR = 0.95, 95% CI: 0.92–0.98) proved to be independent predictors of hyperglycemia. After OGTT, 30 min insulin was lower in hyperglycemic patients (74.2 ± 49.7 versus 94.5 ± 53.9 *μ*IU/mL, *P* = 0.02), while 90 min insulin (170.1 ± 84.6 versus 122.9 ± 97.7 *μ*U/mL, *P* = 0.01) and 120 min insulin (164.0 ± 101.2 versus 85.3 ± 61.9 *μ*IU/mL, *P* < 0.01) were higher. *Conclusions*. NAFLD patients with higher BMI, lower HDL-C, or older age were more likely to have impaired glucose metabolism. An OGTT could be of value for early diagnosis of DM among this population.

## 1. Introduction

Non-alcoholic fatty liver disease is increasingly being identified in routine clinical practice [1]. Although the natural history of this disorder is variable, the presence of non-alcoholic fatty liver disease may lead to an adverse prognosis for some patients, due to liver-related disorders, as well as cardiovascular morbidity and mortality [2–4]. The pathogenesis of non-alcoholic fatty liver disease has been closely related to insulin resistance; this disorder frequently co-exists with impaired glucose tolerance (IGT) or type 2 diabetes mellitus [5–7]. The latter conditions, if present, have been associated with more severe liver disease and unfavorable prognosis [8–11]. 

Screening patients with non-alcoholic fatty liver disease for impaired glucose regulation or type 2 diabetes mellitus could help the earlier diagnosis and treatment of these conditions preventing their possible complications, such as cardiovascular diseases. The measurement of fasting plasma glucose is considered as the preferred initial screening test for the identification of hyperglycemia [12]. The sensitivity of this test can be variable depending on the population evaluated [13, 14]. However, it has not been clearly established, which of the patients with non-alcoholic fatty liver disease should further undergo an oral glucose tolerance test for the above-described purposes. In this regard, we sought to identify predictor characteristics for the presence of impaired glucose regulation or type 2 diabetes in patients with non-alcoholic fatty liver disease and assess for potential differences in these patients regarding glucose metabolism.

## 2. Methods

We prospectively studied a cohort of patients who presented with elevated levels of serum aminotransferases at the outpatient hepatology clinic of Attikon University Hospital, in Athens, Greece, between June 2006 and September 2009, and was diagnosed with non-alcoholic fatty liver disease. The diagnosis of non-alcoholic fatty liver disease was based on the presence of hypertransaminasemia along with characteristic findings of fatty infiltration in liver ultrasonography (“bright liver” or hyperechoic appearance) and the exclusion of other possible causes of hypertransaminasemia, including alcoholic liver disease, adverse events of drugs, viral hepatitis, autoimmune disorders, and hereditary diseases affecting the liver.

The patients that were diagnosed with non-alcoholic fatty liver disease underwent an oral glucose tolerance test, unless they had a known prior history of diabetes or a fasting serum glucose ≥126 mg/dL. Specifically, 75 g of glucose were administered orally after an overnight fast. Serum glucose and insulin were measured in blood samples obtained through an indwelling peripheral vein cannula at time 0 and 30, 60, 90, and 120 min after the glucose challenge. Patients were classified as having normoglycemia (normal glucose values), impaired fasting glucose (IFG), IGT, or type 2 diabetes, according to the criteria endorsed by the American Diabetes Association [15]. Approval for the study was granted by the institutional review board.

### 2.1. Data Analysis

We grouped the non-alcoholic fatty liver disease patients into normoglycemic and hyperglycemic (IFG, IGT, or type 2 diabetes). We assessed for the presence of differences between the above 2 groups in the patient demographics, body mass index (BMI), and common laboratory tests. We also assessed for differences between the 2 groups in the levels of glucose and insulin obtained at the 5 time points specified above during the 75 g oral glucose tolerance test and the overall insulin response during the test. The overall insulin response was determined on the basis of the area under the curve (AUC) of insulin levels versus time, extracting the area corresponding to the baseline insulin level (net incremental insulin AUC). We used the trapezoid method to calculate the AUC. Finally, we calculated the updated homeostasis model assessment of insulin resistance (HOMA2-IR) index and assessed for differences between the 2 groups [16]. We used the independent samples *t*-test and the *x* ^2^-test to compare continuous and categorical variables, respectively. The variables of baseline patient characteristics with different distributions between the normoglycemic and hyperglycemic groups were entered in a stepwise, forward, binary logistic regression model to test for independent associations. *P* values lower than 0.05 were considered statistically significant. We used the SPSS version 15.0 (SPSS Inc., Chicago, ILL) as the software for the statistical analysis.

## 3. Results

**Figure 1:**
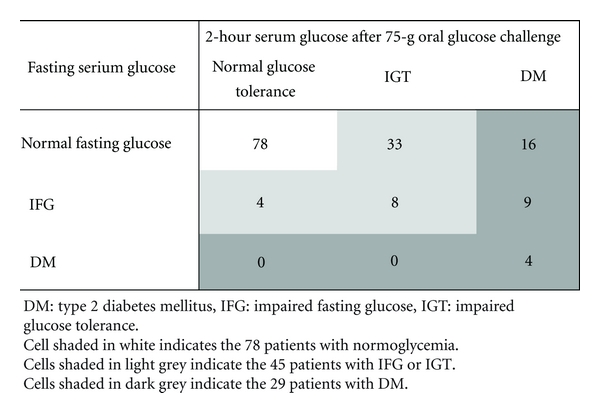
Classification of the 152 included patients with non-alcoholic fatty liver disease on the basis of fasting serum glucose and 2-hour post-load serum glucose.

A total of 152 Caucasian patients (52.0% females and 48% males) with non-alcoholic fatty liver disease, with mean ± standard deviation age of 50.1 ± 11.4 years were included in the study. According to the values of fasting serum glucose or serum glucose 2 hours after the 75 g oral glucose challenge, 78 of the 152 patients (51.3%) were normoglycemic, while 45 patients (29.6%) had IFG or IGT (21 and 41 patients, resp.), and 29 (19.1%) had type 2 diabetes (Figure 1).

In Table 1, we describe the baseline patient characteristics and common laboratory tests in non-alcoholic fatty liver disease patients with or without hyperglycemia. Age, BMI, high-density lipoprotein cholesterol (HDL-C), and serum albumin were the only variables that significantly differed between the 2 studied groups. In the multivariate analysis that included the above four variables as covariates, age (odds ratio: 1.08, 95% confidence interval: 1.03–1.13), BMI (odds ratio: 1.12, 95% confidence interval: 1.01–1.25), and HDL-C (odds ratio: 0.95, 95% confidence interval: 0.92–0.98) were found to be independently associated with the presence of hyperglycemia in patients with non-alcoholic fatty liver disease. Patients with hyperglycemia had greater age (53.0 ± 10.7 versus 47.3 ± 11.4 years, *P* < 0.01), higher BMI (30.5 ± 4.5 versus 28.5 ± 4.8 kg/m^2^, *P* = 0.01), lower HDL-C (46.5 ± 13.6 versus 53.7 ± 18.8 mg/dL, *P* = 0.02), and lower serum albumin (4.1 ± 0.5 versus 4.4 ± 0.4 g/dL, *P* < 0.01) in comparison to patients with normoglycemia (Figure 1).

Table 2 depicts the associations of serum glucose and insulin values obtained at time 0 and 30, 60, 90, and 120 min after the 75 g oral glucose load between non-alcoholic fatty liver disease patients with or without hyperglycemia. Patients with hyperglycemia (IFG, IGT, or type 2 diabetes) had higher glucose values at all the above-specified time points compared with patients without hyperglycemia. Additionally, patients with hyperglycemia showed significantly higher insulin levels at time 0, 90, and 120 min compared with patients without hyperglycemia. Insulin at 30 min was lower in the patients with hyperglycemia, and insulin at 60 min did not differ between the 2 groups. The overall insulin response (net incremental insulin AUC) did not differ, as well. The HOMA2-IR index significantly differed between the two groups (Table 2).

## 4. Discussion

Almost half (48.7%) of the patients with non-alcoholic fatty liver disease who were evaluated in our study and had IFG, IGT, or type 2 diabetes. This group was of older age had higher BMI, lower levels of HDL-C, and lower serum albumin compared with the group of patients without hyperglycemia. Age, BMI, and HDL-C were independent predictors of the presence of IFG, IGT, or type 2 diabetes in our cohort of patients with non-alcoholic fatty liver disease. The patients with hyperglycemia seemed to be more insulin resistant compared with those without hyperglycemia. The acute phase of insulin response to the 75 g oral glucose load was less pronounced in the hyperglycemic group, as evidenced by insulin at 30 min. This was followed by more pronounced hyperinsulinemia at 90 and 120 min after the glucose challenge.

Our study findings agree with those of other studies that have showed variability in the glucose regulation of patients with non-alcoholic fatty liver disease [10]. This could be in part attributed to the fact that non-alcoholic fatty liver disease comprises a spectrum of disorders of different severity, from simple hepatic steatosis to non-alcoholic steatohepatitis, hepatic fibrosis, and cirrhosis [6]. 

In our study, older age was associated with a greater likelihood for impaired glucose regulation in patients with non-alcoholic fatty liver disease. Type 2 diabetes mellitus is known to be associated with older age, a fact that reflects the long process for clinical onset of diabetes mellitus [14]. It can be assumed that metabolic derangements associated with non-alcoholic fatty liver disease and insulin resistance [17, 18] impose a stress on pancreatic *β*-cells that may eventually fail to compensate for the increased insulin requirements. In our study, the acute insulin response to the oral glucose load, which can be considered as a marker of *β*-cell function [19, 20], was found to be decreased in non-alcoholic fatty liver disease patients with hyperglycemia.

Higher BMI is also a known risk factor for type 2 diabetes mellitus [21]. In our study higher BMI independently predicted the risk for the presence of hyperglycemia among patients with non-alcoholic fatty liver disease [17]. More than half of our study population had a BMI below the threshold of 30 kg/m^2^ though. Therefore, our findings should not be interpreted as if only obese patients with non-alcoholic fatty liver disease have hyperglycemia.

Serum levels of HDL-C were also lower in the subgroup of patients with hyperglycemia of the non-alcoholic fatty liver disease patients evaluated in our study. Low HDL-C is a characteristic finding in diabetic dyslipidemia and is also associated with prediabetes, insulin resistance, metabolic syndrome, and non-alcoholic fatty liver disease [20, 22–24]. The elevated levels of serum triglycerides, another feature of diabetic dyslipidemia, did not seem to differ between the non-alcoholic fatty liver disease patients who had hyperglycemia compared with those who did not, in our study. Also, comparison of the two groups on the basis of the ratio triglycerides/HDL-C, a parameter that has been associated with insulin resistance and atherogenicity [25, 26], showed only a marginal difference (Table 1).

Additionally, in our study, the non-alcoholic fatty liver disease patients with hyperglycemia had lower levels of serum albumin compared with those without hyperglycemia, although this difference was not independent of age, BMI, and HDL-C. Presumably, lower serum albumin in patients with non-alcoholic fatty liver disease could also be related to more advanced liver disease, higher degree of systemic inflammation, or albuminuria due to diabetic nephropathy [8, 27]. 

Screening for hyperglycemia could be considered in patients with non-alcoholic fatty liver disease given the strong association of this disorder with IGT, including diabetes mellitus. There are known risk factors for type 2 diabetes to guide the selection of patients to screen [12]. Age above 45 years and the association of a BMI above 25 kg/m^2^ with HDL-C below 35 mg/dL are among the indicators for screening for type 2 diabetes proposed by the American Diabetes Association [12]. In our study, age, BMI, and HDL were independent indicators for the presence of impaired glucose regulation in patients with non-alcoholic fatty liver disease who had no known derangement in glucose metabolism.

If our patients had undergone assessment of fasting serum glucose alone, 25 of the 74 (33.8%) patients with diabetic or non-diabetic hyperglycemia and 4 of the 29 (13.8%) patients with, specifically, diabetic hyperglycemia would have been identified. In contrast, 70 of the 74 (94.6%) patients with diabetic or non-diabetic hyperglycemia and all of the 29 patients with, specifically, diabetic hyperglycemia were identified on the basis of the 2-hour serum glucose value obtained after an oral glucose tolerance test (Figure 1). Considerable discordance in the accuracy of fasting and 2-hour postload glucose for the identification of impaired glucose regulation has been observed in different populations [13, 28]. Fasting glucose may have lower sensitivity in younger individuals [13]. Additionally, IFG and IGT may more accurately reflect hepatic and muscle insulin resistance, respectively [14]. Yet, non-alcoholic fatty liver disease is associated with both hepatic and peripheral insulin resistance [29–31]. 

The oral glucose tolerance test is thought to be rather cumbersome to perform in everyday clinical practice [14]. Our study indicates that non-alcoholic fatty liver disease patients of older age, higher BMI, and lower HDL-C should be prioritized in this regard. In two other similar studies, older age and higher BMI have been associated with abnormal oral glucose tolerance in young male non-alcoholic fatty liver disease patients in China [32], and lower HDL-C has been associated with diabetes mellitus in non-alcoholic fatty liver disease patients in Hong Kong [33]. Besides, increased age and BMI are risk factors for more severe disease or adverse prognosis in patients with non-alcoholic fatty liver disease [2, 8, 9]. 

According to our findings, the value of an oral glucose tolerance test, if performed in patients with non-alcoholic fatty liver disease with appropriate risk factors, would be first to identify those with diabetic hyperglycemia. This group should be managed as for type 2 diabetes, if the diagnosis is confirmed with a second positive relevant test. An oral glucose tolerance test in selected patients with non-alcoholic fatty liver disease can also identify those with impaired glucose tolerance, which indicates an increased risk for developing type 2 diabetes. Although the risk for developing type 2 diabetes can also be determined by considering patient characteristics that are more readily available [34], the presence of impaired glucose regulation signals additionally an increased risk for the development of macrovascular complications [35]. Non-alcoholic fatty liver disease patients with impaired glucose tolerance should be encouraged to modify lifestyle factors (e.g., lose weight and increase physical activity) to prevent the development of type 2 diabetes [14]. These measures would be also important for preventing the progression of liver disease [36]. 

Some of the patients with impaired glucose tolerance and additional risk factors for type 2 diabetes could also be candidates to receive pharmacologic therapy, particularly with metformin [14]. Pioglitazone might also be effective in this regard [37]. These insulin-sensitizing agents have additionally shown promise for the treatment of biopsy-proven non-alcoholic steatohepatitis in small clinical trials [38, 39]. Whether non-alcoholic fatty liver disease patients with impaired glucose tolerance represent appropriate candidates to receive such medications, on top of lifestyle modification, with the aim to prevent progression into type 2 diabetes and to non-alcoholic steatohepatitis and, also, to decrease the overall cardiovascular risk, requires further study.

In conclusion, in our study of patients with non-alcoholic fatty liver disease, older age, higher BMI, and lower serum levels of HDL-C independently predicted the presence of hyperglycemia (defined as IFG, IGT, or type 2 diabetes) that was primarily identified through an oral glucose tolerance test. Conclusively, we suggest that oral glucose tolerance testing should be considered for patients with non-alcoholic fatty liver disease with one or more of the above-mentioned predictor factors for hyperglycemia to readily diagnose and manage disorders of glucose metabolism.

##  Conflict of interests

The authors declare that they have no conflict of interests.

## Figures and Tables

**Table 1:** Baseline characteristics of non-alcoholic fatty liver disease patients with and without hyperglycemia.

Patient characteristics	IFG/IGT, *n* = 45	DM, *n* = 29	Hyperglycemia (IFG/IGT or DM), *n* = 74	Normoglycemia, *n* = 78	*P* value (patients with hyperglycemia versus normoglycemia)
n/N (%) OR mean ± standard deviation					
Sex, Female	21/45 (46.7%)	14/29 (48.3%)	35/74 (47.3%)	44/78 (56.4%)	0.26
Age, years	51.2 ± 10.2	55.9 ± 11.0	53.0 ± 10.7	47.3 ± 11.4	**<0.01**
Body mass index, kg/m^2^	30.0 ± 4.2	31.1 ± 4.9	30.5 ± 4.5	28.5 ± 4.8	**0.01**
AST, U/L	43.7 ± 46.6	38.8 ± 23.5	41.7 ± 38.8	40.2 ± 33.4	0.81
ALT, U/L	64.6 ± 45.9	59.8 ± 39.1	62.7 ± 38.8	67.7 ± 55.6	0.55
AST/ALT ratio	0.68 ± 0.22	0.72 ± 0.30	0.70 ± 0.25	0.71 ± 0.39	0.85
GGT, U/L	86.7 ± 70.8	89.6 ± 87.1	87.9 ± 77.2	86.6 ± 88.7	0.92
Cholesterol, mg/dL	209.4 ± 40.7	209.2 ± 50.8	209.3 ± 44.6	211.9 ± 42.3	0.74
Triglycerides, mg/dL	135.1 ± 64.5	146.4 ± 76.2	139.7 ± 69.1	123.1 ± 72.3	0.18
HDL-C, mg/dL	47.6 ± 14.3	45.0 ± 12.7	46.5 ± 13.6	53.7 ± 18.8	**0.02**
LDL-C, mg/dL	128.3 ± 32.1	131.2 ± 40.3	129.6 ± 35.8	133.9 ± 34.1	0.51
Triglycerides/HDL-C ratio	3.6 ± 3.4	3.6 ± 2.2	3.6 ± 2.9	2.7 ± 2.2	0.05
Total Protein, g/dL	7.0 ± 1.5	7.0 ± 1.6	7.0 ± 1.5	7.1 ± 1.7	0.83
Albumin, g/dL	4.2 ± 0.4	4.0 ± 0.5	4.1 ± 0.5	4.4 ± 0.4	**<0.01**
Albumin/globulin ratio	3.1 ± 7.9	1.8 ± 1.7	2.5 ± 6.1	2.3 ± 3.1	0.83
Hemoglobin, g/dL	14.4 ± 1.5	13.6 ± 1.7	14.0 ± 1.6	14.1 ± 1.4	0.93
Hematocrit, %	43.4 ± 4.6	41.8 ± 4.2	42.7 ± 4.5	42.2 ± 3.8	0.45

ALT: alanine aminotransferase, AST: aspartate aminotransferase, DM: type 2 diabetes mellitus, IFG: impaired fasting glucose, IGT: impaired glucose tolerance, GGT: gamma-glutamyl transpeptidase, HDL-C: high-density lipoprotein cholesterol, IFG: impaired fasting glucose, and IGT: impaired glucose tolerance.

**Table 2:** Glucose and insulin regulation in response to a 75 g oral glucose tolerance test in non-alcoholic fatty liver disease patients with and without hyperglycemia.

Patient characteristics	IFG/IGT, *n* = 45	DM, *n* = 29	Hyperglycemia (IFG/IGT or DM), *n* = 74	Normoglycemia, *n* = 78	*P* value (patients with hyperglycemia versus normoglycemia)
Mean ± standard deviation					
Glucose 0 min, mg/dL	89.9 ± 14.1	104.0 ± 21.9	95.4 ± 18.7	80.9 ± 10.5	**<0.01**
Insulin 0 min, *μ*IU/mL	15.4 ± 8.3	19.5 ± 10.2	17.0 ± 9.3	13.8 ± 7.2	**0.02**
Glucose 30 min, mg/dL	160.4 ± 27.0	179.9 ± 42.1	167.7 ± 34.6	147.8 ± 32.5	**<0.01**
Insulin 30 min, *μ*IU/mL	79.5 ± 53.4	65.3 ± 42.3	74.2 ± 49.7	94.5 ± 53.9	**0.02**
Glucose 60 min, mg/dL	204.5 ± 34.9	241.7 ± 40.3	219.1 ± 41.1	152.9 ± 39.0	**<0.01**
Insulin 60 min, *μ*IU/mL	133.1 ± 72.0	112.4 ± 70.7	125.0 ± 71.7	136.7 ± 88.8	0.38
Glucose 90 min, mg/dL	196.9 ± 37.4	264.1 ± 38.9	219.8 ± 49.4	126.9 ± 32.9	**<0.01**
Insulin 90 min, *μ*IU/mL	163.6 ± 80.0	183.0 ± 95.1	170.1 ± 84.6	122.9 ± 97.7	**0.01**
Glucose 120 min, mg/dL	163.2 ± 24.2	243.6 ± 38.3	194.7 ± 49.8	105.4 ± 21.7	**<0.01**
Insulin 120 min, *μ*IU/mL	160.9 ± 96.3	168.8 ± 110.1	164.0 ± 101.2	85.3 ± 61.9	**<0.01**
Net incremental insulin AUC, *μ*IU/mL * min	11872.9 ± 6608.2	10545.7 ± 6688.5	11352.8 ± 6626.1	10285.4 ± 6690.6	0.33
HOMA2-IR index	1.9 ± 1.0	2.5 ± 1.3	2.2 ± 1.2	1.7 ± 0.9	**<0.01**

AUC: area under the curve, HOMA2-IR: homeostasis model assessment of insulin resistance, DM: type 2 diabetes mellitus, IFG: impaired fasting glucose, IGT: impaired glucose tolerance, and LDL-C: low-density lipoprotein cholesterol.
